# Health-promotion in the context of ageing and migration: a call for person-centred integrated practice

**DOI:** 10.5334/ijic.1162

**Published:** 2014-03-03

**Authors:** Qarin Lood, Synneve Dahlin Ivanoff, Lisen Dellenborg, Lena Mårtensson

**Affiliations:** Institute of Neuroscience and Physiology, Department of Clinical Neuroscience and Rehabilitation, The Sahlgrenska Academy, University of Gothenburg, Sweden; Centre for Person-Centred Care (GPCC), University of Gothenburg, Gothenburg, Sweden; Institute of Neuroscience and Physiology, Department of Clinical Neuroscience and Rehabilitation, The Sahlgrenska Academy, University of Gothenburg, Sweden; Centre for Person-Centred Care (GPCC), University of Gothenburg, Gothenburg, Sweden; Institute of Health and Care Sciences, The Sahlgrenska Academy, University of Gothenburg, Gothenburg, Sweden; Vårdalinstitutet, The Swedish Institute for Health Sciences, Universities of Lund and Gothenburg, Sweden; Institute of Neuroscience and Physiology, Department of Clinical Neuroscience and Rehabilitation, The Sahlgrenska Academy, University of Gothenburg, Sweden

**Keywords:** healthcare inequities, immigration and migration, qualitative research, implementation, integrated care, person-centered care

## Abstract

**Objective:**

For the aim of improving the implementation of a health-promoting intervention for older persons who are born abroad, this study aimed to explore health care professionals' experiences of facilitators and barriers for their possibilities to support a healthy ageing in the context of migration.

**Methods:**

Qualitative data were collected from four focus groups with health professionals who all had experience of working with older persons who are born abroad. Data were analysed with the guidance from the method developed by Krueger and Casey, progressing from an empirical to an abstract level.

**Results:**

Five different conditions were found to influence supporting healthy ageing in the context of migration: *Sense of belonging through significant others, Emotional bonds to a place called home, Expectations on health and support during the ageing process, Mutual understanding as a means for communication* and *Heterogeneity as a point of departure*. The one comprehensive theme *complexity* describes how those aspects are interrelated in a complex and unpredictable way.

**Conclusions:**

The results point at the need for focusing on each person's experiences and health expectations, and the study provides a foundation for future research on the integration of whole-system and person-centred practice.

## Introduction

With increases in the amount of older persons who are born abroad [1], there is a growing need to attend to the issue of equitable health care service delivery. Therefore, with a previous intervention programme as a stand point [2], this study is part of the implementation process of a multidisciplinary health-promoting intervention for older persons who have migrated to Sweden. Both migration status and age seem to be predictors of unequal access to health care services [3], and there is a need for conscious implementation strategies and efforts in order to guarantee that the positive effects of the source intervention remain when implemented in another context [4]. When it comes to persons who are born abroad, there is a notion that health care delivery services are especially important to address [3, 5, 6], with research pointing at serious health care inequities based on country of birth, ethnic origin, religion or preferred language [3, 7–10].

As migration involves a process of social change, it might result in a loss of enabling factors to maintain daily activities and health [3, 7, 11, 12], and it might challenge how older persons experience health during the ageing process [11]. Older persons who are born abroad are at high risk for developing poor health. They face multiple risks of marginalisation, being both old and born abroad [13, 14], and they may experience ageing out of place even years after migration [15]. Even if many health care inequities that older persons who are born abroad experience can be explained by socioeconomic factors, serious inequities regarding access, quality and utilisation of health care services still remain after taken such factors into account [16].

Research points at the need to address attitudes and preconceptions among health care providers when it comes to providing equitable healthcare to a diverse population [14]. Health professionals’ attitudes have previously been reported to influence the provision of health care services to persons who are born abroad [11, 17, 18] and older persons [19, 20]. However, health is a human right [21], and in Europe people who have migrated generally are entitled to the same formal rights as their native-born counterparts [22]. Equitable health care calls for equal access to, and utilisation of high-quality services for equal needs [23]. Nevertheless, the knowledge about how the health of older persons who are born abroad can be promoted is scant [8, 24]. Just a few studies have reported on how to actually implement and assess interventions designed to improve access and uptake, and focus has mostly been on linguistic barriers, such as cost of translation services [25]. Hence, more research is needed in order to dig deeper into how we can overcome barriers to health care access for persons who are born abroad [26].

Organised applications of clinical research findings into routine practice can help improve the quality of health care services [27], but lack of attention to implementation processes makes it difficult to determine how to develop interventions [4, 28, 29]. Providing effective and appropriate interventions that are sustainable over time requires active and planned efforts to make changes to organisations, teams and individual persons [29]. It is important to attend to the different cultures of the organisation providing care, as well as to identify facilitators and barriers within the health care system where the implementation will take place [30]. Even if health professionals have been reported to have negative attitudes towards persons who are born abroad [17], their experiences are crucial to the implementation process as professional actions might influence both development and implementation of interventions [4, 30]. Therefore, the aim of the present study was to explore health professionals’ experiences of facilitators and barriers for their possibilities to support a healthy ageing in the context of migration. In order to capture other relevant aspects of the wide-range process of implementation, the next step will be a qualitative interview study with older persons who are born abroad, which results will be presented in a separate publication.

## Methodology

### Study design

Focus groups were employed to generate qualitative data on the research subject, and to explore the current views of health and ageing from different perspectives [31–33]. The study sought for the experiences of health professionals who meet older persons who are born abroad in their work.

Focus group methodology uses group interaction to give participants opportunities to stimulate each other in discussions in order to explore new issues that arise [32]. The methodology is applicable when exploring areas with limited knowledge [33], and hence relevant in the present study. In order to stimulate the discussion and assist the groups to express different features of the topic, a sample of participants with similar experiences was the objective. However, heterogeneity was also required to ensure variance and to broaden the discussions [31, 32]. This allowed the researchers to gain insight into the participants’ experiences and framework of understanding [31, 32].

The focus group methodology of the present study is based on basic assumptions from social constructivism, with the assumption that people interact and are influenced by each other [34]. Through group interaction, the participants of a focus group discussion challenge each other's points of view and new knowledge can be constructed. Moreover, focus group discussions can make participants aware of things they would never have thought of otherwise. Hence, participants learn from each other and from their interactions the method endorses a collective understanding of the world [31, 34].

### Study setting

Data collection took place at the participants’ workplaces, located in a suburban area of a middle-sized city in Sweden. A large proportion of persons who are born abroad inhabit the suburb, and the choice of the study setting was based on requirements for the venue for the focus group to be relaxed and comfortable [33]. Therefore, the authors were also careful to ensure that the room was informally set up, in an area without disturbances from outside.

### Recruitment of participants

Eighteen health professionals (social workers, home help professionals, registered occupational therapists, physiotherapists and nurses) were recruited through managers in primary and municipality care units. Information regarding the project was sent out to the managers and if they decided that the unit should participate in the study they signed a consent form and forwarded the information to health professionals whom they considered as potential participants according to the inclusion criteria: health professionals with experience of working with older persons who are born abroad. This shared experience ensured homogeneity in the focus groups, and to further certify homogeneity participants were divided by professional field. Occupational therapists and physiotherapists were considered to belong to the same professional field and participated in the same focus group discussion. In order to also ensure heterogeneity, purposive sampling [35] was used in order to access a variety of perspectives according to length of work experience, work place and gender. Professionals who agreed to participate signed an informed consent form and more information was provided to them orally before the focus group discussions started. The regional ethical review board in Gothenburg, Sweden, granted formal ethical approval (Reg nr: 821-11).

### Data collection and analysis

A topic guide was developed by the authors in order to form a structure for the group discussions. The topic guide was based on the content of the source intervention, and aimed to cover health professionals’ experiences of working with older persons who are born abroad in relation to potential facilitators and barriers for health care provision. The last author moderated all focus group discussions, with support from the first author who followed the discussions as an observer and wrote field notes regarding the participants’ behaviour and interaction. Both were experienced health care professionals, and the moderator had extensive experience of leading focus groups. After each focus group discussion, the first author listened to the recording and took notes on immediate impressions in order to deepen the question areas for the next focus group. All focus groups lasted for approximately one hour, were recorded digitally and then transcribed verbatim by the first author (the three first focus groups) and a professional transcription company (the final focus group).

All recordings were listened to several times and the transcriptions were continuously re-read throughout the analysis process which was guided by the qualitative method described by Krueger and Casey [33]. Notes were written down in the margin of the transcription, and the analysis took on a long-table approach in order to identify themes and categories. This low-technology approach involves cutting the transcribed data apart into sections of text that answer to the aim of the study [33]. The first author colour-marked each section in order to be able to identify which focus group they came from, and then all sections were spread out on a big wall to get an overview over the condensed data, and to identify themes and categorise the results. Sections of text answering to the same questions were sorted together in preliminary categories, which were then discussed among the authors repeatedly until the final categories and theme were identified. In order to reach a qualitative formulation, all authors interpreted the meanings of the condensed data, cautious not to lose the context in the interpretation procedure. Finally, the qualitative formulations were compared with the raw data to ensure that the categories and themes disclosed the underlying meanings in the text.

## Results

In total, four focus groups were conducted with 4–5 participants per group. Most of the participants were female (15 out of 18) and were born in Sweden (13 out of 18). Their work experience differed between 3 and 21 years (see Table 1 for demographical characteristics of the participants). The analysis resulted in five categories describing health professionals’ experiences of facilitators and barriers for their possibilities to support healthy ageing in the context of migration. The complex and unpredictable interrelations between the categories are described in one comprehensive theme. Descriptions of categories and theme are presented in running text and quotations are used to clarify their content. Brackets and parentheses are used to put the quotations in context.

### Sense of belonging through significant others

The category visualises how sense of belonging to significant others was experienced by the professionals to both facilitate and hinder the provision of health care services to older persons who are born abroad. Sense of belonging was described to involve reciprocity, feelings of fellowship and respect, and older persons who are born abroad were experienced to rely on inter-reliant relationships to people to whom they feel a sense of belonging. Predominantly, this referred to compatriots and close family members whom were experienced as a resource for the older persons, and facilitators for the provision of health care services. However, the discussions on sense of belonging also mirrored ambivalence, with the professionals reflecting upon how sense of belonging may also become a barrier if older persons develop a dependency on significant others with whom they feel a sense of belonging to. The professionals therefore emphasised the need for interactions between the older person, his or her significant others and the health professional when planning health care interventions.

P2: And then for a lot of reasons, but maybe mainly because one wants to stay safe within the family, and can apply for home help benefit, the younger generation go in and give their support so one possibly has difficulty in seeing outside or to actually *know* that there are other solutions. There's something that can make life easier, and that it's there as soon as you can, can get in…

P1: Yes, if you get to know about it in time.

P2: And to, so to speak, inform, intervene. Yes, precisely.

P1: Then you can influence an opinion, too, I think.

P2: Right.

P1: Not just that it just happens even when we come in: it might be that you've made your mind up that it should be only within the family.

P4: Yes.

P1: And that no one else *can* help.

P2: No

P1: We actually understand this at times, too. And we try to motive home help services, and that you get to see that it's not always the best thing to have help from family either. But of course if you can get in early and somehow explain that…

(Group 2, social workers)

### Emotional bonds to a place called home

The category describes the professionals’ experiences of how a psychological feeling of home where the older person feels safe and in control is important to consider when providing health care services. Frequent travel to the country of birth was experienced as a means for the older persons to handle their health, and acknowledging this resource for health was expressed as an important facilitator for health care provision. The professionals shared the experience that older persons who are born abroad often feel better, during as well as after, visits to the country of birth. However, strong attachments to the country of birth were also experienced to involve emotional detachment from the new country and a barrier to health care access. As a means to overcome such barriers, the professionals emphasised the importance of acknowledging the older persons’ maintenance of an emotional bond to the country of birth.

P3: Yes I've heard from kids like that their mum can hardly walk when she's at home [in Sweden], but as soon as we arrive [in the home nation] she runs round the houses, nattering away. These shaky legs – I don't get it, she can hardly move in Sweden.

P5: That's important, too.

(Group 1, occupational therapists and physiotherapists)

### Expectations on health and support during the ageing process

Involving the older persons’ understanding and beliefs of their situation, expectations on health and support during the ageing process were experienced as both facilitators and barriers for health care access. The professionals experienced older persons who are born abroad to expect a poorer health status with age, challenging how they handle health-related issues. An acceptance of symptoms was described as a barrier to health care access and might result in the older persons seeking care too late. Furthermore, not knowing what support to expect from the health care system in the new country might impede the older persons’ possibilities and motivation to participate in and utilise different interventions. As a response to this, the professionals experienced how they might need to accept that older persons who are born abroad expect an increased amount of support from close relatives during the ageing process.

P2: Then a bit like – if they're afraid of taking their own responsibility for their ailment and treatment or… if it's a cultural thing to expect outside help, I don't know.

P4: Expectations, yes, I… it's a part of their culture that the family looks after its own. And then go so far that they get sick and then get lots of diagnoses when they do come to us, and the relatives are maybe tired.

P2: They're not seeking help in time so they can get…

P4: Yes, they don't come in time.

P3: I've felt that they don't take their diagnoses seriously, their ailments and so on either. That it all sorts itself out, sort of.

(Group 3, nurses)

### Mutual understanding as a means for communication

Mutual understanding was experienced as an important means for communication between older persons who are born abroad and health professionals, and as a facilitator for health care provision. Mostly, mutual understanding referred to linguistic understanding of words, and the importance of a shared language permeated almost all parts of the focus group discussions. However, the professionals also emphasised the need to build respectful and trustful relationships as a means to achieve a mutual understanding. Openness and flexibility were considered as superior to linguistic understanding when it comes to building such relationships that can improve the older persons’ possibilities to know what to expect from health care services in the new country.

P5: They don't want to get involved. And they think that “ah, I can't speak the language, I can't be part of it”. But there are resources available, like…

Moderator: But do you all mean that it hangs on the language?

P5: No, their own will too.

Moderator: How do you mean?

P1: And the culture.

Moderator: What do you mean, culture?

P1: They've grown up in a different way.

P5: We had a lady, nearly 90. She cried when she couldn't go out and meet people, and she didn't speak the language. She knew no Swedish whatsoever but she went and sat with Swedish people every day, and if she couldn't get out one day she'd cry. It's all so different.

Moderator: But if you're saying they're different, which ones would you say it would be best to invest these preventative measures in? Would you care to say something about that?

P2: Well, those who are closest to these people. Or if they themselves could seek help at an early stage.

(Group 4, home help professionals)

### Heterogeneity as a point of departure

Heterogeneity was considered as an important aspect to consider, addressing each person's different personal characteristics and previous life experiences. Even if the professionals expressed specific means for older persons to age healthily in the context of migration, they also experienced older persons who are born abroad as a heterogeneous group, both similar and different to their native-born counterparts. The necessity of addressing each person's unique needs and attitudes was emphasised, and predicting a person's needs or interpretations of information based on country of birth or migration status was considered as impossible and inappropriate. Therefore, as opposed to make generalisations, the professionals expressed a need to attend to each person's resources and strengths when providing health care services.

P3: In terms of experience, those who arrived early [migrated a long time ago] have fallen into our regulatory system and they know they are entitled to rehabilitation and come in that way. The others who arrived as relatives and so forth are very difficult to catch outside of the family, and to do anything beyond the home environment, like, it's hard to get them out from their home.

…

P4: Sure, I think it's so difficult to differentiate…

P4: Yes, it's very hard.

P4: … groups, now I'm thinking about groups…

Moderator: I understand.

P5: Yes it is.

P4: And they're actually different, all of them, it's different for those in this group too, it really is.

P5: Yes.

P4: But as you say, it's hard [to get them out from the home].

Moderator: What's your experience? You can take examples, even if they're different. It can be more generalised than you think…

P4: Yes, sometimes I think that it can be that way [for persons who are born abroad], but it may be the same for other groups too.

(Group 1, occupational therapists and physiotherapists)

### Complexity

The theme visualises an ambivalence and variation in the professionals’ discussions on their experiences of working with older persons who are born abroad. The professionals struggled with the complexity of how everything affects everything, and no facilitator or barrier could be regarded as more important than another. Even if the professionals sometimes described how one specific category could influence health care provision, it was more common that they described the categories as interrelated and overlapping. Aspects in one category could function as both a facilitator and a barrier, depending on influence from other aspects within the same or other categories. The professionals found it impossible to explain or predict how, when or why different aspects interact and they did not believe in implementation of a generalised intervention for a specific group of people. Even if the professionals expressed specific means for older persons who are born abroad to access health care services, they were ambiguous regarding what influence migration status might have on facilitators and barriers. Instead of focusing on migration status or country of birth, the professionals experienced a need to attend to each person's resources and acknowledge what might facilitate or hinder health care access for different people.

## Discussion

The aim of this study was to explore health professionals’ experiences of facilitators and barriers for their possibilities to support a healthy ageing in the context of migration. Elaborating on the present results, it is clear that there is no intervention that fits all persons. The findings visualise health-promotion for older persons who are born abroad as complex and influenced by several interrelated aspects described in five different categories. Characteristics associated with migration may play a role when implementing health-promoting interventions for older persons, but focus should be on each person's unique needs and the findings call for person-centred integrated practice.

The present study represents one part of the implementation process of a health-promoting intervention for older persons who are born abroad. The findings will be used in order to put focus on how to facilitate collaboration between health professionals and the persons that the services are aimed at. Put in relation to implementation literature, this is relevant as professional actions and team function might influence the implementation process [4]. Through the theme ‘complexity’, the present findings illustrate how a person-centred approach could facilitate health care provision. The professionals’ response to the complexity of providing health care services for older persons who are born abroad was to acknowledge each person's experiences of what might facilitate or hinder health care access. This is clearly related to the person-centred literature, describing how there is a need to proceed from each person's narratives in order to provide high-quality care [36, 37]. Narratives refer to how the person describes his or her current situation and previous experiences, and they rely in interaction between the narrator and the listener [37], commonly described as a partnership [36]. Partnerships require collaboration and continuity of care [37], and they are a prerequisite for shared decision-making. Each person should feel that his or her feelings, beliefs, experiences and preferences are equally important as professional knowledge and experiences [36, 38, 39].

The scientific literature has also visualised a need for health care services to be both person-centred and integrated in order to meet the needs of older persons [40, 41]. Kodner [41] has described a need for health care services to be integrated in order to meet the needs of an ageing population, and with focus on clinical coordination and continuity, multidisciplinary teams could allow for effective evaluation and development of interventions [41]. Johansson et al. [42] have further emphasised the need for integrated multidisciplinary actions when providing health care to older persons in the community [42]. Integration with focus on coordination within a specific health care system could facilitate health care provision and improve both quality and satisfaction with health care provision [43]. However, research on interprofessional collaboration often lacks the person-centred perspective, failing to acknowledge the persons whom health care services are aimed for [44]. In the context of migration, the findings visualise how a person-centred approach can be used to avoid stereotypes based on country of birth. Those findings are confirmed by Björk-Brämberg and Nyström [45] who have previously highlighted the need to attend to each person's life experiences and points of view instead of focusing on cultural aspects when providing health care to persons who are born abroad [45]. A person-centred approach is a prerequisite for equitable health care service provision, with its view on the health care professional and the person seeking care as equal partners of the team [36].

The present findings visualise specific aspects that might facilitate or hinder health care provision for older persons who are born abroad, independently or in concert. The categories ‘sense of belonging through significant others’, ‘emotional bonds to a place called home’ and ‘mutual understanding as a means for communication’ describe health professionals’ experiences of what might be migration-specific facilitators and barriers for health care provision. Those results are strengthened by previous research [46], describing sense of belonging and bonds to a common environment as a recurrent emotional response to forced displacement [46]. Furthermore, mutual understanding has also been a clear focus within the migration research field, and linguistic differences between health professionals and persons seeking care are often visualised as a barrier that might result in poorer-quality care [47], higher costs [48] and lower utilisation [49]. However, the present findings also describe how linguistic differences alone cannot explain why barriers to health care access exist. The category ‘mutual understating as a means for communication’ visualises the importance of respect towards each other, and how the health professionals experienced trustful relationships and linguistic understanding as equally important. Hadziabdic et al. [50] have also described the importance of focusing on other aspects than linguistic understanding when providing health care to persons who are born abroad [50]. The theme ‘complexity’ provides additional information of how facilitators might become barriers, depending on how they are influenced by each other and the surrounding context. In relation to Brown's [51] application of complexity theory to health care contexts, health-promotion for older persons who are born abroad can be understood as being influenced by facilitators and barriers that constantly influence, and adapt to, each other and their context in an unpredictable way. As a response, the category ‘heterogeneity as a point of departure’ visualises the professionals’ experiences of how health care provision should be based on personal narratives, rather than country of birth or migration status. Hence, in contrast to previous research, focusing on migration status as a determinant for healthcare provision [17, 18, 52–54], the present findings describe professional experiences of how country of birth cannot explain or foresee what might facilitate or hinder healthcare provision. Instead the professionals emphasised the need to attend to how facilitators and barriers are experienced by each person in order to reach the goal of equitable health care services.

The present findings visualise a need for person-centred integrated practice when providing health care to older persons who are born abroad. However, integrating scientific knowledge into previous health care practices is a complex challenge for health care organisations. A suggestion for future research is therefore to further explore how to implement person-centred integrated health-promotion for older persons who are born abroad.

### Key lessons for action

- Be aware that older persons who are born abroad are a heterogeneous group. Disregard pre-existing assumptions of their situation and social context in favour for their own story.
- Enable shared decision-making through relations where all parts are listened to, feel respect for each other and are allowed to tell their story.
- Make time to develop well-functioning multidisciplinary teams, which have the possibility to attend to a variety of health-related issues.
- Make sure that the older person whom the multidisciplinary intervention is intended for is considered as an equal member of the team.

### Methodological strengths and limitations

Focus group methodology provides possibilities to diminish researcher control through a focus on the group and interactions between participants. Moreover, the shared disclosure between participants can make it possible to include participants who would have been reluctant to take part in an individual interview [33].

Regarding credibility, discussions with experienced researchers in the field and within qualitative research enhanced the quality of the results. Every effort was made to certify that the data extractions were not taken out of context, or were interpreted to suit the authors’ preconceptions. The authors acknowledged the potential influence of the perspective as health care professionals might have had on the results, and the third author complemented with a social anthropological perspective during the review and discussions of the analysis process and emerging interpretations.

Dependability was ascertained through using the same topic guide for all focus groups, and through discussions between the authors regarding similarities and differences in the data. Finally, a clear and distinct description of study context, selection and characteristics of participants, data collection and analysis process enhances the transferability of the findings.

Internal logic was strived for through letting the aim of the study guide the choice of data collection and analysis method. Regarding richness of content, constant comparisons with raw data reflect the professionals’ experiences in an unbiased manner. In harmony with the richness of content, the quality requirement of a clear structure with a reduction of complicated descriptions was in focus throughout the analysis procedure [55].

Concerning the validity of the results [35], quotations are used in order to strengthen the empirical anchoring of the findings. The quotations contribute to a consistency [55] between the interpretation of the data as a whole, and of distinct parts of the data. All experiences of participants quoted in the findings are interpreted and presented in a truthful manner, but some quotations may be experienced as detached and difficult to understand since they have been taken out of their context; consequently, brackets were used in some quotations in order to make the context clearer.

High levels of validity and reasonableness [35] were ensured through the choice of focus group methodology [33]. However, an acknowledged challenge is the risk of encouraging a uniformity of views, inhibiting some participants to express themselves [56, 57]. In order to minimise this risk, the groups were composited with regard to both heterogeneity and homogeneity. Dividing the participants by professional field helped to ensure homogeneity, but to also cover the diversity within the participants they were selected to represent both genders when possible, different ages, workplace and length of work experience.

Participants were selected primarily to illuminate the topic of interest and it was the richness of the collected data that finally made the sample size definite [31]. It is possible that a greater number of participants could have affected the richness of content as more diverse experiences could have been explored, for instance, if other professional groups had been invited to participate. However, the choice of venues for the focus group discussions helped to increase the richness of the content, as they were familiar and comfortable environments for the professionals. After four focus group discussions with a diversity of health professionals, richness of the content was considered as satisfying and the material as saturated [35].

Finally, qualitative methodology does not aim for generalisability, but for a deeper understanding of a certain phenomena. Hence, the transferability of the findings is important to discuss. The participants all worked in the same geographical area, which could be interpreted as a limitation. However, considering the choice of geographical area with a high proportion of older persons who are born abroad in relation to the aim of the study, the limitation was considered as inferior to the advantages of the study setting. Even so, it is important to remember that the findings cannot be extended to other contexts without elaboration [56]. Therefore, a clear and distinct description of study context, selection and characteristics of participants, data collection and analysis process is provided in order to enhance the transferability of the findings. This study adds to the scientific knowledge a deeper understanding of what health professionals experience as barriers and facilitators in health-promotion for older persons who are born abroad. However, qualitative findings must always be understood with regard to the context and specific sample of the study [35], and it would be of interest to carry out further research to explore how health professionals in another geographical location experience the study subject.

## Conclusion

The results provide new data on how health professionals experience facilitators and barriers when providing health care to older persons who are born abroad. This acquired knowledge is relevant for implementation research, providing information on how health-promotion for older persons who are born abroad can be understood as a complex adaptive system, and what agents might be important to address in order to provide equitable health care services. In order to reach this goal, health care providers need to collaborate and deliver services according to narratives and holistic needs of each person. This calls for the implementation of person-centred integrated practice, but more research is needed in order to gain knowledge on how to move from knowledge to action.

## Figures and Tables

**Table 1.**
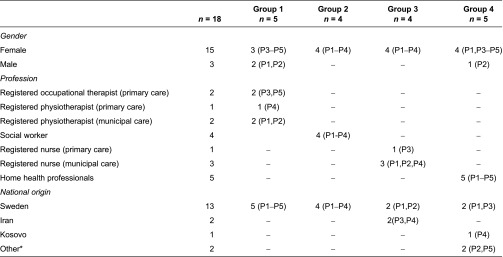
Demographical characteristics of participants (*n*=18)

*Those participants did not choose to reveal their national origin.
